# Neutrophil extracellular traps in hepatocellular carcinoma are enriched in oxidized mitochondrial DNA which is highly pro-inflammatory and pro-metastatic

**DOI:** 10.7150/jca.64170

**Published:** 2022-01-24

**Authors:** Lu-Yu Yang, Xiao-Tian Shen, Hao-Ting Sun, Wen-Wei Zhu, Ju-Bo Zhang, Lu Lu

**Affiliations:** 1 Department of General Surgery, Huashan Hospital, Fudan University, Shanghai, China; 2 Caner Metastasis Institute, Fudan University, Shanghai, China; 3 Liver disease center, Huashan Hospital, Fudan University, Shanghai, China; 4 Department of Infection Disease, Huashan Hospital, Fudan University, Shanghai, China

**Keywords:** Neutrophil extracellular traps, hepatocellular carcinoma, oxidized mitochondrial DNA, metastasis

## Abstract

**Background:** Neutrophil extracellular traps (NETs) are net like extracellular structure formed by neutrophils in response to certain stimulation. It works as inflammatory regulator and metastasis promoter in cancer. Mitochondrial-(mt)DNA is a circular, mitochondria derived double strain molecule, which is involved in NETs formation. Its role in NETs induced inflammatory alteration in hepatocellular carcinoma (HCC) remained unexplored.

**Method:** We evaluated the mitochondrial reactive oxygen species (mitoROS) level in peripheral neutrophils from HCC patients and the oxidative level of mtDNA in derived NETs. The association between the NETs and oxidized mtDNA was assessed to reveal their relevance. A function assay was applied to uncover how the oxidation state of mtDNA directed the metastasis promoting inflammation state in HCC cells in a NETs protein dependent manner. Finally, using animal models, we explored the potential of a therapy strategy against NETs-drove metastasis by targeting the oxidized mtDNA with metformin.

**Results:** Neutrophils in HCC patients contained high level of mitoROS level, and formed NETs that were enriched in oxidized mtDNA in a mitoROS dependent manner. NETs and oxidized mtDNA were clinically relevant. Bound with NETs protein, oxidized mtDNA is more capable of triggering the metastasis-promoting inflammatory mediators in HepG2 cells. Targeting the oxidized mtDNA with metformin attenuated the metastasis-promoting inflammatory state and hereby undermine the metastasis capacity of HCC.

**Conclusion:** HCC is capable to stimulate NETs enriched in oxidized mtDNA, which are highly pro-inflammatory and pro-metastatic. Oxidized mtDNA in NETs may serve as a potential anti-metastatic target by metformin therapy.

## Introduction

Neutrophils play a pivotal role in the acute phase of the inflammatory response and in host defense 1, 2. Increasing evidences highlight the plastic role of neutrophils in cancer, where they can be polarized toward distinct phenotypes. An anti-tumor N1 phenotype and are able to kill tumor cells, while a pro-tumor N2 phenotype sustains genetic instability, tumor growth, and metastasis 3-5.

In addition to releasing proteolytic enzymes and inflammatory mediators, neutrophils also extrude three-dimensional mesh called neutrophil extracellular traps (NETs) 1, 4. NETs are made up of chromatin as backbone and a number of proteins including peptides with antimicrobial properties such as myeloperoxidase, neutrophil elastase (NE) and LL37, an antimicrobial peptide of the cathelecitin family 6, 7. NETs are first discovered as host defense to restrict infection, and are then implicated in autoimmune diseases, sterile inflammation, thrombosis. In addition, emerging reports have suggested NETs affected tumor progression and metastasis through multiple possible mechanism. In our previous work in hepatocellular carcinoma (HCC) 8, we found that NETs activated Toll-like receptor (TLR) signal, and trigger an metastatic-supporting tumorous inflammatory response featured as elevation of cyclooxygenase (COX)2 and other inflammatory mediators. The metastatic promoting NETs can be triggered by several mediators in cancer setting. Indeed, we and others have observed a similar trend of increased NETs formation from cancer associated neutrophils 9-12. Candidate NETs-promoting mediators include G-CSF, CXCL8/IL8 in different cancer models. Despite the increased NETs formation, how cancer structurally alters NETs are less elucidated.

Mitochondria, by generating reactive oxygen species (mitoROS), plays multiple roles in energy metabolism and cellular homeostasis 13, 14. Mitochondrial DNA (mtDNA) is a circular double stranded DNA molecule. Compared to nuclear DNA (nDNA), mtDNA is substantially more prone to oxidative stress, and is highly pro-inflammatory as a more potent TLR ligand 15, 16. MtDNA is also detected in NETs, suggesting mitochondrial involvement in NETs formation 17-19. Generation of mitoROS in neutrophils is critical for NETs formation. The host cells mitochondrial activity and metabolic process are often altered by cancer.

In this study, we reported that neutrophils in HCC patients contained high level of mitoROS level, and formed NETs that were enriched in oxidized mtDNA in a mitoROS dependent manner. A clinical relevance of NETs, mtDNA and oxidative stress were assessed. Through functional assay, we further uncovered that the bound mtDNA potentialized HCC neutrophils-extruded NETs more potent to trigger the metastasis-promoting inflammatory mediators in trapped HCC cells.

## Results

### NETs from HCC neutrophils are enriched in mtDNA and highly oxidized

**Figure 1:**
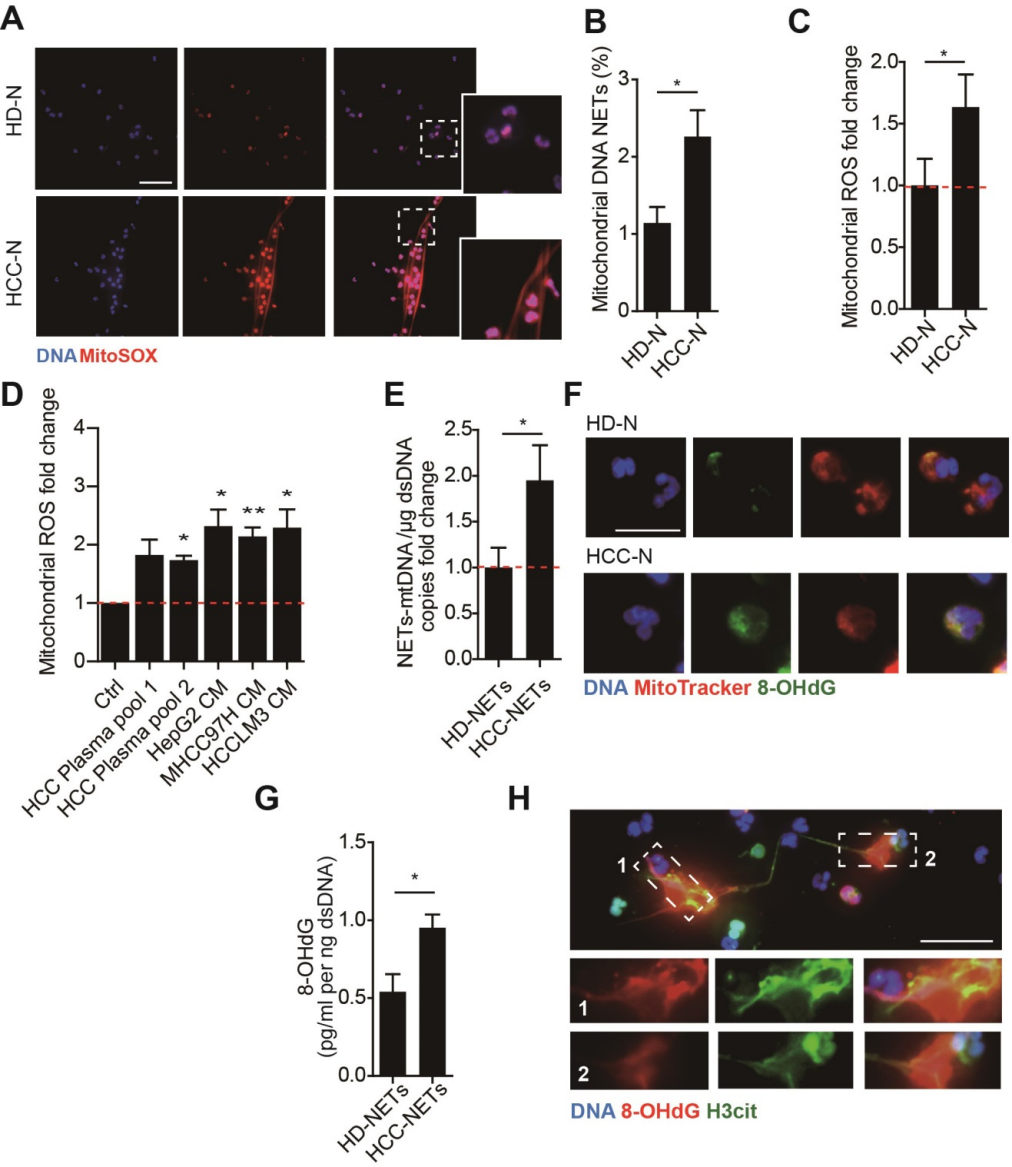
** Higher mitochondrial ROS and oxidized mtDNA are released with the enhanced NETs formation in HCC-N. (A-B)** Representative immunofluorescence images (A) of enhanced mtDNA (MitoSOX) released with the spontaneous NETs formed by HCC-N (n=13) compaired to HD-N (n=13), and quantification of mtDNA-0Ucontaining NETs (B). Scare bar: 20μm. **(C)** Mitochondrial ROS level in isolated HCC- N (n=13) and HD- N (n=13). **(D)** Higher mitoROS level in isolated HD-N incubated in HCC plasma pool or HepG2/MHCC97H/HCCLM3 CM. **(E)** Mitochondria DNA level in NETs formed from HCC-N (n=13) and HD-N (n=13). **(F)** Representative immunofluorescence images of higher stain of 8-OHdG in mitochondria of neutrophils. Scare bar: 20μm.** (G)** Higher level of 8-OHdG in isolated HCC- (n=13) and HD-NETs (n=13).** (H)** Representative immunofluorescence images showing 8-OHdG stain within HCC-NETs. * P<0.05.

We first investigated the potential presence of mtDNA in NETs from HCC neutrophils (HCC-NETs). In line with our previous report, immunofluorescence suggested that peripheral neutrophils from HCC patients underwent enhanced spontaneous NETs formation in vitro. Here we further determined the formed NETs from HCC neutrophils were enriched in mtDNA as revealed by MitoSOX staining of mitoROS content within the web-like NETs structure **(Figure 1A-B)**. As release of mtDNA is driven by cellular mitoROS, we found HCC neutrophils consistently contained higher level of intracellular mitoROS **(Figure 1C)**, which explained the presence of mtDNA in NETs. In comparison, normal neutrophils from HD were relatively static, as we observed fewer spontaneous NETs formation and low level intracellular mitoROS **(Figure 1A-C)**. Incubating normal neutrophils in HCC plasma or condition medium (CM) from HCC cell lines HepG2, MHCC97H and HCCLM3 are capable to induce NETs. Supporting these results, HCC plasma and CM of HCC cell lines were found to upregulate mitoROS level in neutrophils, and prime normal neutorphils towards NETs formation, suggesting certain tumor environmental factors alter neturophils mitochondrial activity **(Figure 1D)**. To better illustrate the enrichment of mtDNA in tumorous NETs, we performed PCR assay to further detect mtDNA in total chromatin content from PMA-stimulated NETs, and revealed a higher portion of mtDNA copy number in NETs from HCC neutrophils compared to that from HD **(Figure 1E)**. These jointly suggested HCC NETs were enriched in mtDNA. Moreover, a co-localization of MitoTrack and oxidation indicator 8-OHdG under fluorescent immunostaing suggested a highly oxidation of mitochondria content in HCC neutrophils **(Figure 1F)**. Consistently, NETs-DNA extruded from HCC neutrophils were also highly oxidized as detected by 8-OHdG level, a marker of oxidation **(Figure 1G-H).** Collectively, these results indicated that HCC neutrophils were more primed towards NETs formation, and the HCC-NETs were enriched in mtDNA and highly oxidized compared to normal NETs.

### NETs are associated with mtDNA oxidation in HCC patients

**Figure 2:**
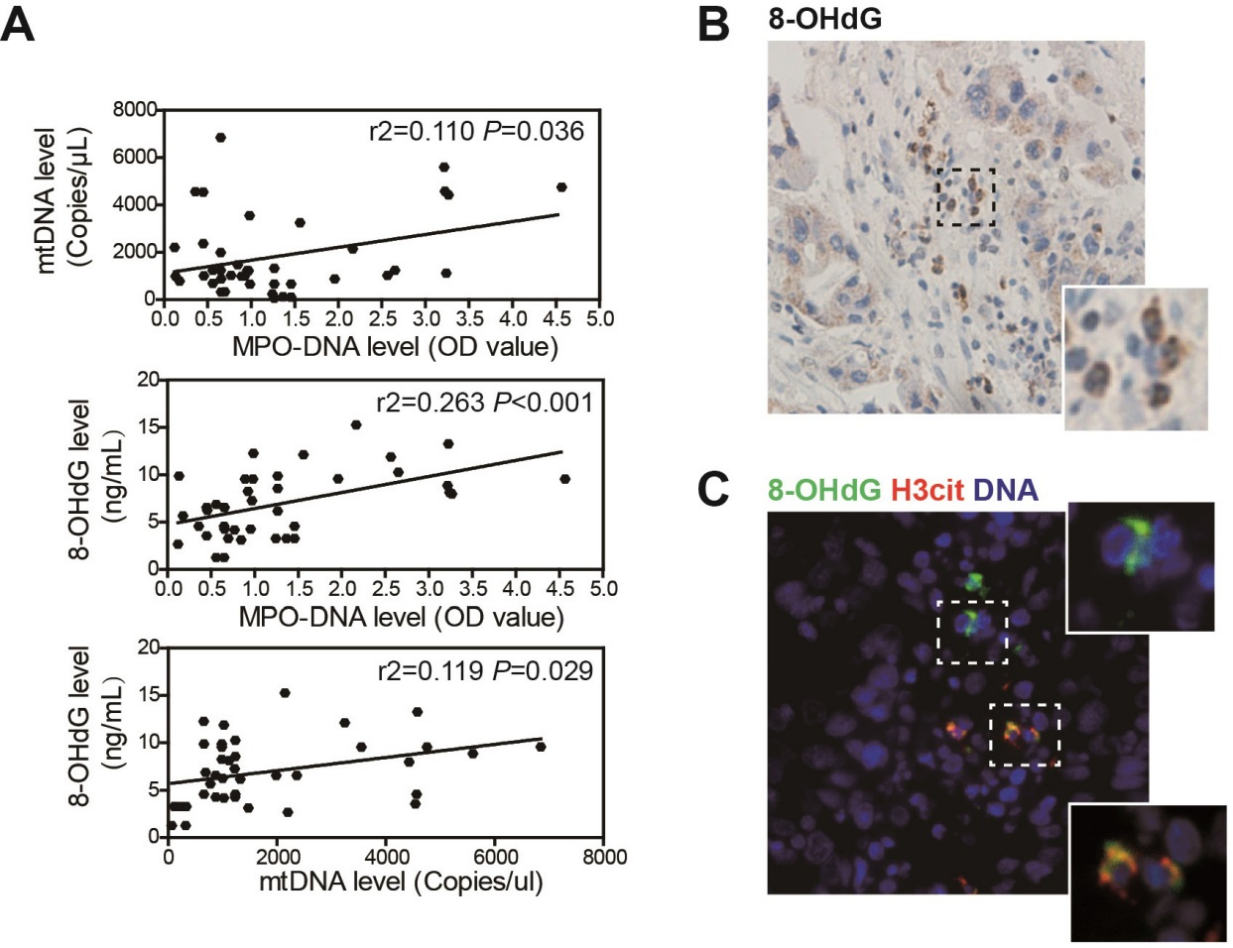
** NETs is correlated with a higher oxidized mtDNA level in HCC patients. (A)** Correlation analysis of serum MPO-DNA complex, free mtDNA and 8-OHdG level from HCC patients (n=53) by ELISA. **(B)** Representative immunohistochemistry image of stromal 8-OHdG stain in HCC resected tissue.** (C)** Representative immunofluorescence image of 8-OHdG and H3cit (NETs marker) stain in HCC tissue.

We further assessed the association between NETs, mtDNA and oxidation level in clinical sera samples from 53 HCC patients by ELISA. Circulating NETs marker MPO-DNA level was increased in HCC sera, and this was in correlation with circulating mtDNA copy number, suggesting that the elevated mtDNA level was, at least in part, derived from NETs **(Figure 2A, upper panel)**. As we found HCC NETs were highly oxidized, a correlation between MPO-DNA and circulating 8-OHdG level was also observed **(Figure 2A, middle panel)**. Moreover, as mtDNA are more prone to oxidation, a close correlation between circulating mtDNA copy number and 8-OHdG level was within expectation **(Figure 2A, lower panel)**. Moreover, in serial sections of human HCC samples, we found strong expression of 8-OHdG in infiltrated host cell of polymorphonuclear feature within tumor stromal, suggesting an oxidized state of tumour-infiltrated neutrophils **(Figure 2B)**. In situ immunofluorescence staining further proved the stromal expression of 8-OHdG was co-localized with NETs marker H3cit **(Figure 2C)**. These jointly illustrated the close correlation between NETs and mtDNA oxidation in HCC clinical samples.

### MitoROS is required for the NETs formation in HCC neutrophils

**Figure 3:**
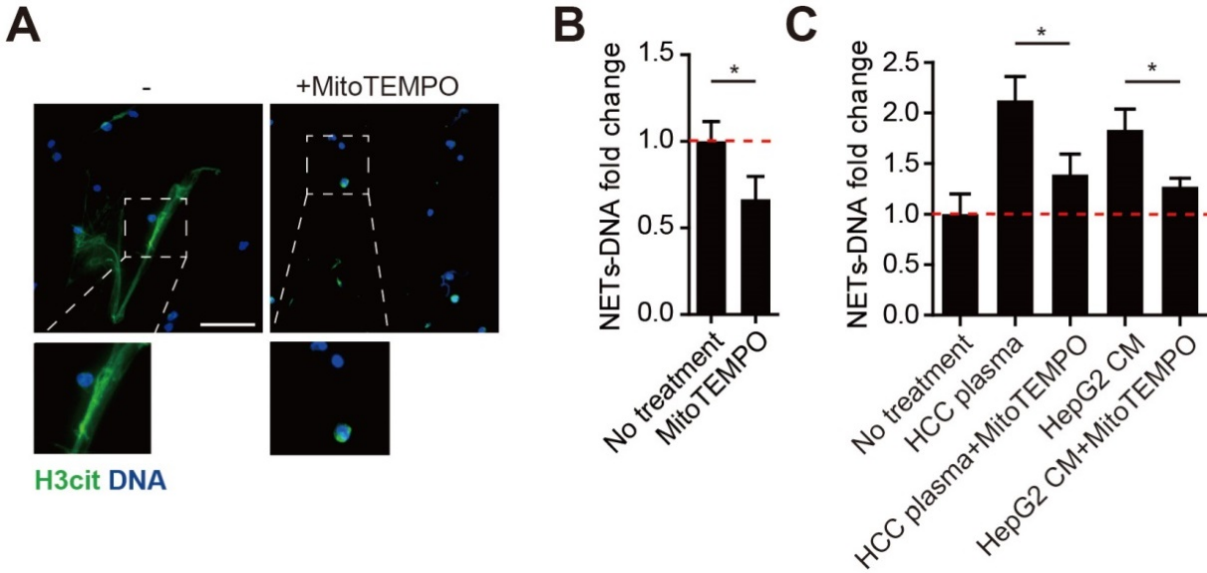
** Mitochorial ROS is required for malignant-associated NETs formation. (A-B)** Representatvie immunofluorescence images (A) and quantification (B) of attenuated NETs formed by HCC-N treated with mitoTEMPO. Scare bar: 100μm. **(C)** NETs-DNA released by normal neutrophils stimulated with HCC plasma or CM from HCC cell lines, and attenuated by MitoTEMPO. * P<0.05.

MitoROS is critical to NETs formation, and abolishing mitoROS reduces NETs in lupus. As we found HCC neutrophils contained high level of mitoROS and extruded mtDNA-enriched NETs, we then investigated the necessity of mitoROS in NETs formation from HCC neutrophils. To our expectation, spontaneous NETs formation of HCC neutrophils was reduced in the presence of specific mitoROS scavenger MitoTEMPO (Figure 3A-B). In line, a similar reduction was observed in NETs from normal neutrophils incubated with HCC plasma or HCC cell lines CM (Figure 3C). These results suggested the elevated mitoROS level in HCC neutrophils served as one possible mechanism of the enhanced NETs in HCC neutrophils.

### HCC-NETs enriched in oxidized mtDNA are more potent inducer of tumorous inflammatory response

**Figure 4:**
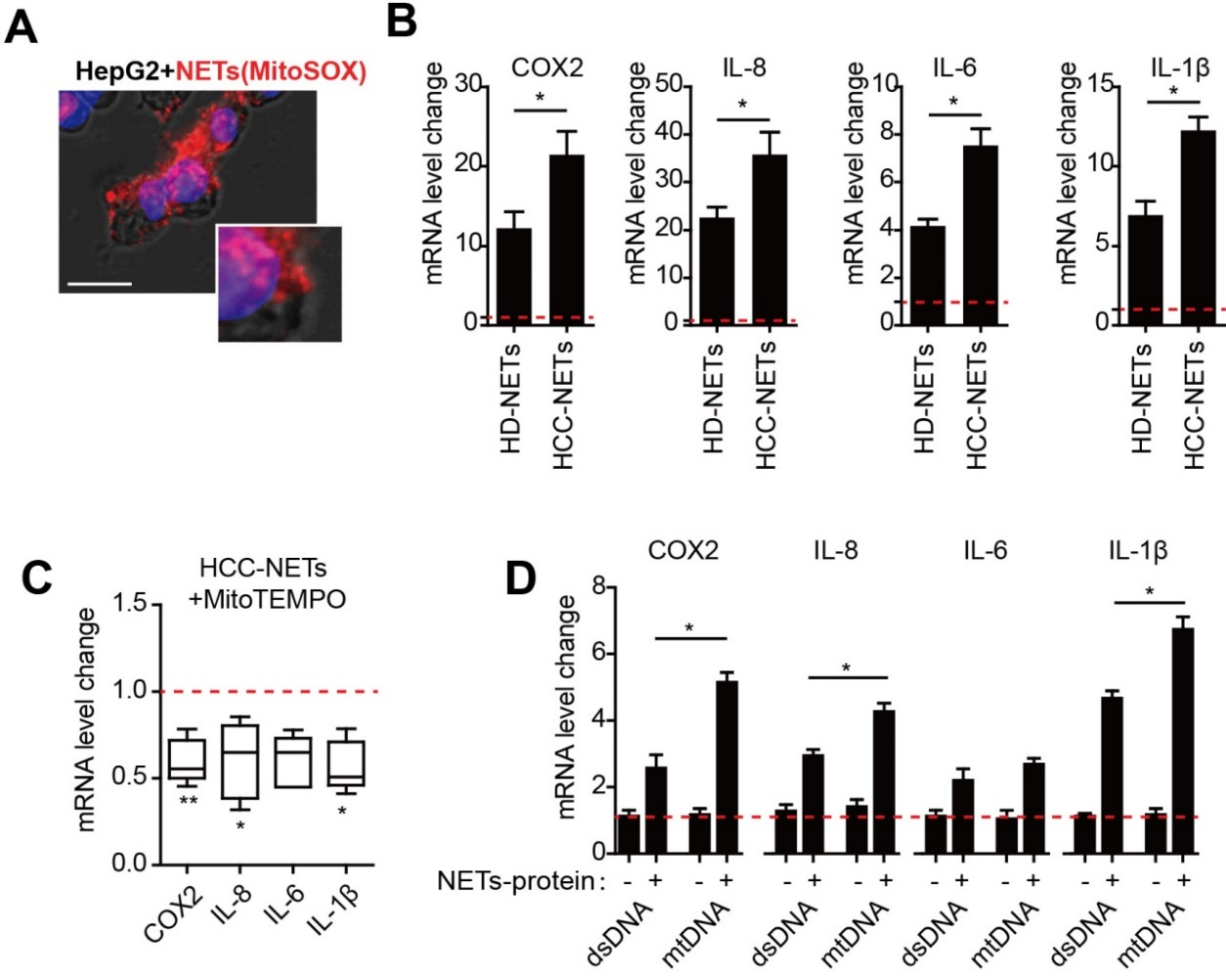
** HCC-NETs enriched in oxidized mtDNA are more potent inducers of tumorous inflammatory response. (A)** Representative image of internalization of NETs into HepG2 cells. NETs was marked with MitoSOX (red), nuclear was contour stained with DAPI.** (B)** The mRNA level of tumorous COX2, IL8, IL6 and IL-1β in an incubation system of HD- or HCC-NETs and HepG2 cells. **(C)** The mRNA level of tumorous COX2, IL8, IL6 and IL-1β in MitoTEMPO-pretreated HepG2 cells stimulated by HCC-NETs. **(D)** The mRNA level of tumorous COX2, IL-8, IL-6 and IL-1β in HepG2 cells stimulated by isolated DNA alone or with NETs-protein. * P<0.05, ** P<0.01.

We have reported that provoked tumorous inflammatory response was the key mechanism to NETs- triggered metastatic potential including invasion and pro-survival capacity in HCC cells 8. As we found NETs from HCC neutrophils were different from normal NETs referred to mtDNA component, we then assessed the possible dissimilarity between HCC- and HD-NETs referred to the NETs-triggered tumorous inflammatory response. In an incubation system of NETs and HepG2 cells, we observed NETs the internalization into HCC cells, as the first step to initiate subsequent tumorous inflammatory response (Figure 4A). Rt-PCR revealed several inflammatory mediators, including COX2, IL8, IL6 and IL-1β, were upregulated in NETs-treated HepG2 cells, and this was more pronounced in the setting of HCC-NETs, as stimulation of HepG2 with HCC-NETs resulted in an enhanced production of the inflammatory mediators (Figure 4B). Since we found HCC-NETs were enriched in mtDNA that was highly oxidized, we then further investigate the underlining mechanism of the enhanced stimulatory effect of HCC-NETs. To our expectation, decreasing mitochondrial oxidation level by anti-oxidant MitoTEMPO partly abrogated the upregulated pro-inflammatory mediators stimulated by HCC-NETs (Figure 4 C). As NETs are composed of both proteins and chromatin from nuclear and mitochondria, we then isolated different NETs components separately to further assessed their stimulatory effect on HCC cells. Free DNA content alone, whether from mitochondria or not, failed to stimulate HepG2 to produce more inflammatory mediators. NETs-protein complexed with mtDNA from HepG2 CM-treated neutrophil-like dHL60 cells in NETs presented stronger stimulatory effect on tumorous COX2, IL8, IL6 and IL-1β, comparing to DNA/NETs-protein complex with a relative moderate stimulatory effect (Figure 4D). These suggested the oxidized mtDNA enriched in HCC-NETs caused the pronounced inflammatory response in HCC cells, which might aid progression and metastasis.

### Metformin attenuate oxidized mtDNA-enriched NETs and reduce HCC metastasis

**Figure 5:**
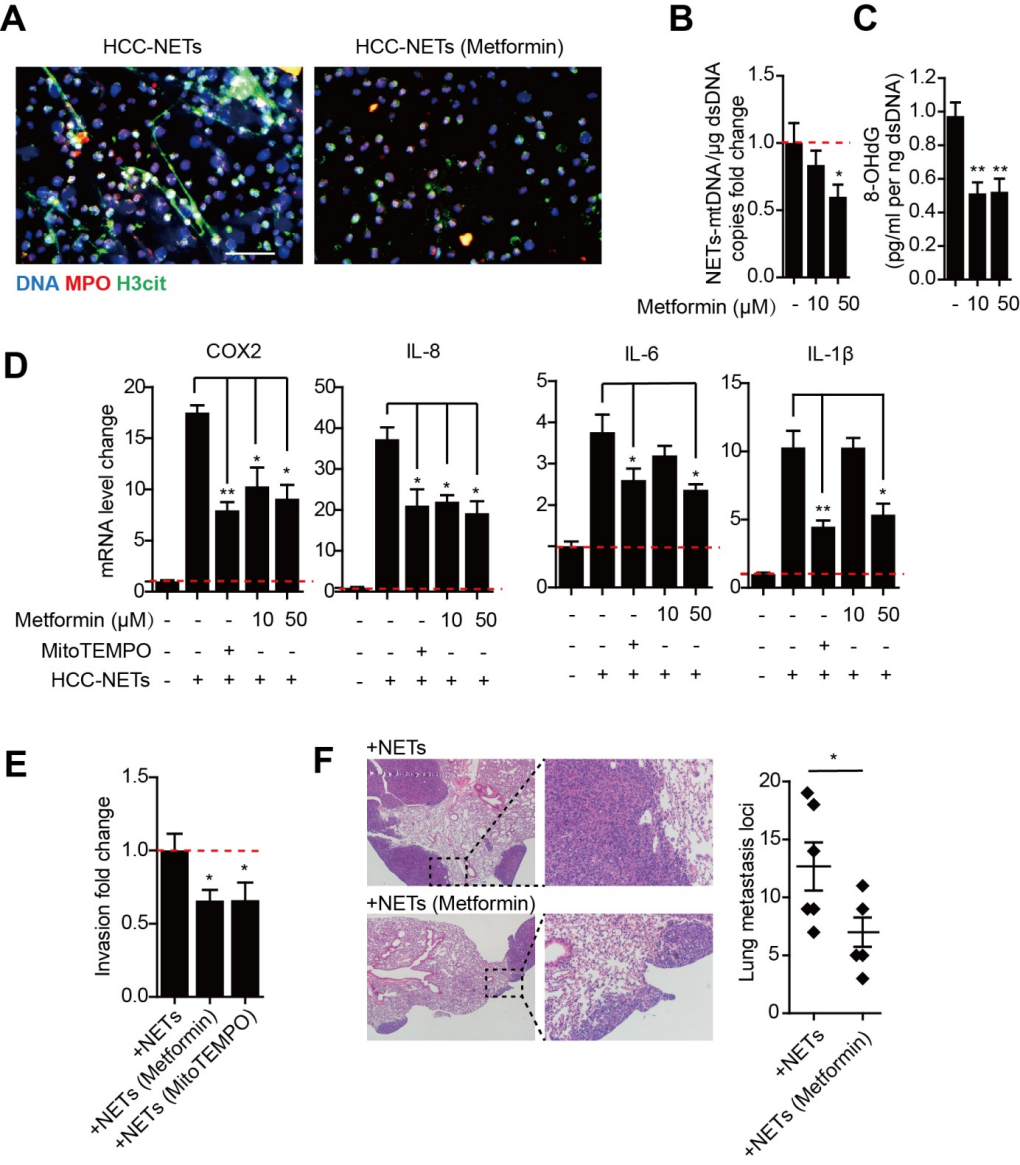
** Diminishing altered oxidized mtDNA-enriched HCC-NETs by metformin reduced tumorous inflammatory response and HCC metastasis. (A)** Metformin inhibited HCC-NETs formation. HCC-N were pretreated with or without metformin and stimulated by PMA for 4 hours. NETs were then fixed and stained for DNA, MPO and H3cit, and representative immunofluorescence images were shown. Scare bar: 100μm. **(B-C)** metformin attuned mtDNA level in NETs (B) and level of 8-OHdG in NETs (C). Data were presented as fold change relative to basal level of each group. **(D)** Metformin attuned the pro-inflammatory mediators up-regulation in HepG2 cells triggered by HCC-NETs. MitoTEMPO served as a positive control for oxidation inhibition. **(E)** Metformin inhibited the enhanced invasion capacity induced by HCC-NETs. **(F)** Metformin abrogated the experimental metastasis in a mice model. Representative of gross lung metastasis and quantification were shown. * P<0.05, ** P<0.01.

Metformin is the mainstay treatment for type 2 diabetes mellitus and has diverse mechanisms of action including anti-oxidative properties and anti-inflammatory effects. The formation of NETs is ROS dependent, while metformin can selectivity inhibit mitochondrial respiratory chain complex I and decrease NADPH oxidase activity, thus leading to a decrease in ROS production. We found treating HCC neutrophils with metformin prior to PMA stimulation reduced NETs formation (Figure 5A). Metformin significantly decreased mtDNA copy number and oxidation indicator 8-OHdG in PMA-induced HCC-NETs (Figure 5B-C). Moreover, metformin diminished the up-regulation of several metastatic-promoting inflammatory mediators triggered by HCC-NETs, which was comparable to anti-oxidant MitoTEMPO as positive control (Figure 5D). We further assessed whether metformin could abrogated NETs and diminished HCC metastasis. The invasion of HCC cells exposed to HCC-NETs was consistently diminished by metformin treatment (Figure 5E). In line with the in vitro result, in a mice model, intravenous administration of HCC-NETs increased HepG2 experimental lung metastasis in mice, and this was also abrogated by metformin (Figure 5F). These findings collectively suggested metformin by reducing oxidized mtDNA in HCC-NETs and attenuating NETs formation, abrogated the inflammatory response provoked by NETs and reduced HCC metastasis.

## Discussion

The emerging role of NETs in cancer has been studied. NETs and intact neutrophils can catch tumor cells and thereby facilitate their adhesion for metastasis. More recently, it was reported that NETs may also awaken dormant cancer cells and suppress anti-tumor T cells 20, 21. Extensive NETs formation has been reported among different studies on various tumor-bearing mice and cancer patients 4, 11, 14. In the present and previous research, we showed a higher enrichment of NETs in HCC patient serum or conditioned medium of HCC cells educated neutrophils, indicating they have a higher capability to form NETs. HCC not only increased the NETs quantity, but also modified its composition by increasing the mtDNA. It is persuasive that neutrophil is responsible for the elevated circulating mtDNA in HCC, as a high correlation of NETs protein and mtDNA is found in the patients sera. In consistence with our study, He et al found that immune cell derived mtDNA is present in HCC patients, and predicted a worse outcome 22.

We and others have found NETs formation in HCC are enhanced in quantity, as more NETs level were detected in isolated HCC patients peripheral neutrophils and clinical sample 8, 23, 24. Here, we further suggested HCC-NETs were different from normal NETs in context of NETs-bounded mtDNA level, as HCC-NETs were enriched in mtDNA that was highly oxidized. Supportive to our findings, intracellular levels of ROS are found higher in monocytes and neutrophils from HCC patients as well as mitochondrial superoxide in neutrophils 25. Moreover, studies have reported leukocyte mtDNA content is associated with risk of several cancers including HCC 22. The altered mtDNA content in HCC neutrophils might be the source and one driving mechanism of the mtDNA-enriched HCC-NETs in the present study. What led to the increase of mtDNA in neutrophils remains less illustrated. It has been suggested that cancer associated neutrophils undergo metabolic alteration and switched from glycolytic to fatty-acid consuming, leading to mitochondrial ROS accumulation and increasing the mtDNA 26, 27. In line, we found an increased ROS generation measured by 8-OHdG in HCC educated neutrophils and NETs mtDNA, indicating that HCC positively regulated the ROS generation in neutrophils, stimulating the formation of oxidative mtDNA enriched NETs, hence harnessing neutrophils to facilitated metastasis. However, whether and how exactly the neutrophil undergo energy switch to increase the ROS generation required further exploration.

As NETs trap HCC cells and further trigger a pro-metastatic tumorous inflammatory response, the altered mtDNA content in HCC-NETs led to a question that whether NETs pro-inflammatory capacity on HCC metastasis was altered by mtDNA content in HCC-NETs. Indeed, mtDNA has long been recognized as an important component of NETs and a pro-inflammatory mediator 28, 29. Extracellular and cytosolic mtDNA is able to activate damage associated molecular patterns (DAMPs) and cause sterile inflammation during liver carcinogenesis 30-32. Moreover, studies revealed a positive relation between oxidation state of mtDNA and its pro-inflammatory capacity in immune disease, indicated that oxidized mtDNA works as a strong pathologic factor 27. Here we found that oxidation state of mtDNA in NETs is decisive to the up-regulation of some inflammation related gene, such as COX2, IL8, IL6 and IL-1β in HCC cells. In line with us, Kitti et al reports that oxidative modification enhances the immunostimulatory effects of extracellular mitochondrial DNA on plasmacytoid dendritic cells to produce more IFNα. One interesting finds was that DNA alone, whether from mitochondria or not, was not sufficient to provoke the tumorous inflammatory response, where NETs protein was required in complex with mtDNA content. Similarly, it has been reported that complexing extracellular DNA with peptides such as LL-37 enhances DNA up-take into living cells in cancers and psoriasis^33^ suggesting that NETs-protein was necessary to the internalization of NETs into HCC cells and the subsequent enhanced activation of tumorous pro-inflammatory signaling by the enriched oxidized mtDNA content in HCC-NETs.

Given the altered mtDNA content was functionally related to the enhanced HCC-NETs formation as well as the metastatic-promoting role of HCC-NETs, abrogating mtDNA content in HCC-NETs served as an appealing strategy against HCC metastasis. Here we showed that metformin treatment with its anti-oxidative and anti-inflammatory capacity, reversed the up-regulation of NETs-induced pro-metastatic tumorous inflammatory response in vitro and attenuated the NETs-induced lung metastasis in a mice model, making metformin work as an effective, low-cost and plug-to-play therapy to prevent the pre-established HCC metastasis.

## Methods

### Human specimens, animals and cell lines

Peripheral blood samples were collected from 53 HCC patients (HCCs) with pathological diagnosis and 13 healthy donors (HDs). Among them, neutrophils were isolated from peripheral blood for 13 HCCs and the 13 HDs for in vitro NETs study. Serums were prepared from all 53 HCC patients for detection of MPO-DNA, mtDNA and 8-OHdG level. Paraffin-fixed surgically resected HCC samples were also collected for NETs detection. All samples were obtained in our institute under the regulation of the Ethics Committee of Huashan Hospital, Fudan University in agreement with the Declaration of Helsinki with written consent. HCC cell lines were cultured in DMEM supplemented with 10% FBS (Gibco), 1% penicillin/streptomycin at 37°C in a 5% CO_2_ incubator. Six- to 8-weeks old of male null-mice were used in animal studies. All animal experiments were approved by the Animal Ethics Committee of Fudan University.

### Isolation of human neutrophils

Neutrophils were isolated from the peripheral blood obtained from HCCs and HDs by a widely used one-step gradient centrifugation method using Polymorph^ Prep^ (Axis-Shield) according to instruction, and resuspended in RPMI 1640 supplemented with 5% fetal calf serum (FCS) for immediate use. A purity of 90% was confirmed by flow cytometry (FACS Calibur) using anti-CD15 antibody staining (BD Bioscience), with a viability rate over 95% by Trypan blue exclusion.

### NETs formation study

To evaluate NETs formation capacity, freshly isolated human neutrophils from HCCs (HCC-N) and HDs (HD-N) were adjusted to a concentration of 5x10^5^ cells/ml and incubated for 4 hours to form NETs in 20nM PMA (Phorbol 12-myristate 13-acetate, Sigma), HCCs plasma pools (1:2) or HCC cell lines conditioned medium (CM) as stimulation, or pretreated with MitoTEMPO (10 µM, Sigma) and metformin (Sigma) 30min prior to stimulation. For visualization of NETs, neutrophils were seeded on poly-L-lysine-coated coverslips in 24-well plates to form NETs in different condition, and then fixed with 4% PFA for 20min. Subsequently, coverslips containing NETs were permeabilized with 0.1% Triton X-100 for 15 minutes at RT, washed with phosphate-buffered saline, and blocked with PBS containing 1% bovine serum albumin for 1 hour at RT. NETs were stained in blocking buffer at 4°C overnight with primary antibodies: MPO (1:100, Abcam), H3cit (1:250, Abcam). After washed with PBS, NETs were stained with fluorochrome-conjugated secondary antibodies (Jackson; 1:600) in blocking buffer and finally stained with Hoechst 33342 (1:1000, Thermo Fisher Scientific) to visualize DNA. Slides were then mounted with Fluoro-gel (Beyotime) and observed under fluorescence microscopy. In some experiments, cell-impermeable DNA dye SytoxGreen (Thermo Fisher Scientific, 1:10000), mitochondria ROS indicator MitoSOX (Thermo Fisher Scientific, 1:2000) and cell-permeable DNA dye Hoechst 33342 (1:1000) were added to the incubation plate according to the manufacturer's direction. At the end of incubation, the plates were directly moved to fluorescence microscope (Leica) for NETs formation visualization. Images were collected and analyzed with ImageJ software. For quantification of NETs-DNA, NETs were formed in 96-well plate and NETs-DNA generated by neutrophils were digested with 500 mU/ml micrococcal nuclease (MNase; Worthington). The nuclease activity was stopped with 5 mM EDTA and the culture supernatants were collected and stored at -80℃ until further use. NETs-DNA in the supernatants was quantified by PicoGreen dsDNA dye (Thermo Fisher Scientific) with fluorescence spectrometry under filter setting of 480 nm/520 nm excitation/emission and semi-quantitatively standardized to control group.

### Immunostaining of 8-Hydroxydeoxyguanosine (8-OHdG) in neutrophils mitochondria

Mitotracker Red (200 nM, Beyotime) was added to the isolated neutrophils seeded on poly-L-lysine-coated coverslips at 37˚C for 30 minutes. The cells were then fixed and stained for 8-OHdG (1:150, StressMarq Biosciences) as described above for visualization of NETs.

### Preparation of NETs and NETs-protein

NETs and NETs-proteins were prepared based on the established methods 8, 34. In brief, 1x10^7^ human neutrophils were stimulated with 20nM PMA for 4 hours. Then the supernatants were discharged carefully by slow suction and washed twice to eliminate residual PMA or NETs-unassociated substances without disturbing NETs. Medium RPMI (1ml) containing MNase (1U/ml, Sigma) was then added to digest NETs from the dish at 37˚C for 20 minutes followed by 5mM EDTA to stop nuclease activity. The supernatant containing NETs were collected and centrifuged to eliminate cell debris. The NETs-proteins were isolated from the supernatant containing NETs by applying DNase 1 (10U/ml, Sigma) to totally wreck DNA content in NETs followed by 5mM EDTA to stop nuclease activity with only NETs-protein remained. Isolated NETs and NETs-proteins were stored at -80°C for further use.

### Measurement of serum MPO-DNA level

We measured MPO-DNA complexes in human and mice serum using a well-adopted capture ELISA assay with some modification. Briefly, as the capturing antibody, 5 µg/ml anti-human MPO antibody (1:100, Abcam) was coated to 96-well plates overnight at 4˚C. After blocking in 1% BSA, 100 µl of diluted serum was added per well and incubated at RT on a shaking device for 2 hours. After washing five times with PBST, PicoGreen® dsDNA Quantitation Reagent was added according to manufacturer's directions. The values were then read with a fluorometer with a filter setting of 480 nm/520 nm excitation/emission and semi-quantitatively standardized to HDs or control group.

### Mitochondria DNA (mtDNA) level detection

The level of mtDNA was quantified using a modified PCR procedure 35, 36. Briefly,total DNA was purified from isolated NETs using a QIAamp DNA Mini kit (Qiagen), and quantified using PicoGreen as above. Then 10 ng DNA was subjected to Real-Time PCR with Power SYBR Green PCR Master Mix (Invitrogen) and specific primers encoding mitochondria gene NADH dehydrogenase subunit 1 (5′-GCATTCCTAATGCTTACCGAAC-3′ and 5′-AAGGGTGGAGAGGTTAAAGGAG-3′). Results were expressed as mtDNA copy number fold change relative to each control group. A similar procedure is adopted to quantify serum mtDNA level.

### 8-OHdG level detection

Level of 8-OHdG was detected using a ELISA assay 37. For detection of 8-OHdG-modified nucleotides, 250 ng NETs-DNA was added to poly-l-lysine-coated plates and incubated at 4°C overnight, washed twice and blocked with 1% BSA for 2 hours at room temperature. Oxidized nucleotides were detected by 8-OHdG antibody (1:100, StressMarq Biosciences) at room temperature, followed by incubation with horseradish peroxidase (HRP)-conjugated streptavidin. The assay was developed with TMB (3,3′,5,5′-tetramethylbenzidine), and absorbance was analyzed at 450 nm. Serum 8-OHdG was detected in similar procedure.

### Tissue Immunohistochemical and immunofluorescence staining

Immunohistochemical staining in paraffin-embedded sections was performed by the avidin-biotin-peroxidase complex method. Briefly, after rehydration and microwave antigen retrieval, primary antibody 8-OHdG (1:150, StressMarq Biosciences) was applied, incubated at 4°C overnight, and followed with secondary antibody incubation (GeneTech) at 37°C for 30 minutes. Staining was performed with 3,30-diaminobenzidine tetra hydrochloride and counterstaining was performed with Mayer's hematoxylin. Immunnofluorescence staining of NETs in resected HCC samples was performed in frozen section by 4% PFA fixation, incubation of primary antibody H3cit (1:150, Abcam) and 8-OHdG (1:150, StressMarq Biosciences), and fluorochrome-conjugated secondary antibody incubation with Hoechst 33342 stain of nuclear.

### Isolation of dsDNA and mtDNA

Mitochondria were isolated from neutrophil-like HL60 cells using a Mitochondria Isolation Kit (Thermo), then the mtDNA was extracted from the isolated mitochondria using a DNeasy Blood & Tissue kit (Qiagen). Double-strand DNA (dsDNA) was extracted from HL60 cells by same procedure. The DNA level was detected using PicoGreen® dsDNA Quantitation Reagent described above.

### Invasion capacity of HepG2 cells* in vitro*

1x10^5^ HepG2 cells in serum-free DMEM were seeded on the upper chamber of 8-μm Transwell system coated with Matrigel (BD) in the stimulation of semi-quantified NETs (source neutrophils to form NETs:HepG2, 5:1). In some experiments HepG2 cells were pretreated with MitoTEMPO (10μM) and metformin (50μM). After 30 hours incubation, the contents of the upper chambers were aspirated, washed and cleared by a cotton swab. Cells on lower membranes were then stained with crystal violet. Cells that invaded through the membrane to the lower surface were quantified in 4 random fields as fold change.

### Quantification of neutrophil ROS

HCC-N and HD-N were isolated and incubated with MitoSOX and results were quantified by plate reader following the manufacturer's instructions.

### Quantitative real-time PCR of proinflammatory genes expression

5x10^5^ HepG2 cells was treated with 1μg isolated DNA alone or in complex with semi-quantified whole NETs or NETs-protein (source neutrophils to form NETs:HepG2, 5:1) for 6 hours in vitro, and then total HepG2 RNA was extracted using Trizol (Invitrogen) and reverse-transcribed into single-stranded cDNA using PrimeScript™ RT Reagent Kit (TaKaRa Biotechnology). Quantitative Real Time PCR was performed with SYBR Green qPCR Master Mix (DBI Bioscience). The following specific primers were provided in **supplementary table**. Expression levels were normalized against β-actin in each sample and then standardized as fold change.

### Animal study

Six-8 weeks old male nude mice were subjected to intravenous injection of HCC-NETs (containing in vitro PMA-stimulated NETs from 1x10^7^ HCC-N for each mouse in 100 μl saline, and followed by NETs-treated 1x10^6^ HepG2 cells intravenous injection in 100 μl saline 20 minutes later. To assess the abrogation of metformin on NETs and NETs-related metastasis, NETs-treated HepG2 cells were pretreated by metformin (50μM) as described above prior to intravenous injection, and meanwhile mice were orally given metformin (200mg/kg) prior to NETs injection and continued per day. Mice were then sacrificed at day 30 and lung metastasis loci were quantified with H&E section.

## Supplementary Material

Supplementary tables.

## Abbreviations

HCC: hepatocellular carcinoma
NETs: Neutrophil extracellular traps
mitoROS: mitochondrial reactive oxygen species
COX2: cyclooxygenase 2
NE: neutrophil elastase
