# Supplementary material for: Neutrophil extracellular traps in hepatocellular carcinoma are enriched in oxidized mitochondrial DNA which is highly pro-inflammatory and pro-metastatic
Source: J Cancer. 2022 Jan 24;13(4):1261–71. doi: 10.7150/jca.64170 (PMC8899377; doi:10.7150/jca.64170)
Supplement: Supplementary file 1 — Supplementary tables. [file jcav13p1261s1.pdf]

Table S1. Clinicopathological characteristics of HCC patients for NETs pathological analysis (n = 53)

Characteristics	Case number	%
Sex		
Female	18	33.9%
Male	35	66.1%
Age (years)		
≤ 55	10	18.9%
> 55	43	81.1%
Preoperative serum AFP (ng/mL)		
≤ 20	18	33.9%
> 20	35	66.1%
HBsAg		
Negative	22	41.5%
Positive	31	58.5%
Liver cirrhosis		
No	33	62.2%
Yes	20	37.8%
Tumor size		
≤ 5cm	37	69.8%
> 5cm	16	30.2%
Tumor number		
Single	47	88.6%
Multiple	6	11.4%
Tumor encapsulation		
None	13	24.5%
Complete	40	75.5%
Vascular invasion		
No	23	43.4%
Yes	30	56.6%

Table S2. Real-time PCR primers used in the study

Primers	Seqeunces (5'-3')
IL-8	F-CTCCAGCCACACTCCAACAGA
	R-CACCCTAACACAAAACACGAT
IL-6	F-CCACGGCCTTCCCTACTTC
	R-CTGTTGGGAGTGGTATCCTCTGT
IL-1β	F-CTAAAGTATGGGCTGGACTG
	R-AGCTTCAATGAAAGACCTCA
COX2	F-GAGAAAACTGCTCAACACCG
	R-GCATACTCTGTTGTGTTCCC
